# Impact of a pharmacist-led medication review on hospital readmission in a pediatric and elderly population: study protocol for a randomized open-label controlled trial

**DOI:** 10.1186/s13063-017-1798-6

**Published:** 2017-02-09

**Authors:** Pierre Renaudin, Karine Baumstarck, Aurélie Daumas, Marie-Anne Esteve, Stéphane Gayet, Pascal Auquier, Michel Tsimaratos, Patrick Villani, Stéphane Honore

**Affiliations:** 1 Hôpitaux de Marseille, Hôpital La Timone, Service Pharmacie, Marseille, F-13000 France; 2 Hôpitaux de Marseille, Hôpital La Timone, Unité d’Aide Méthodologique à la Recherche Clinique et Epidémiologique, Marseille, F-13000 France; 3 Hôpitaux de Marseille, Hôpital La Timone, Service de Médecine Interne, Gériatrie et Thérapeutique, Marseille, F-13000 France; 4 Aix-Marseille Université, Service de Pharmacie Clinique, Faculté de Pharmacie Timone, Marseille, F-13000 France; 5 Hôpitaux de Marseille, Hôpital La Timone, Service de Pédiatrie Multidisciplinaire, Marseille, F-13000 France

**Keywords:** Medication review, Medication reconciliation, Randomized controlled trial, Clinical pharmacy, Pharmacists, Pediatrics, Geriatrics

## Abstract

**Background:**

Early hospital readmission of patients after discharge is a public health problem. One major cause of hospital readmission is dysfunctions in integrated pathways between community and hospital care that can cause adverse drug events. Furthermore, the French ENEIS 2 study showed that 1.3% of hospital stays originated from serious adverse drug events in 2009. Pharmacy-led medication reviews at hospital transitions are an effective means of decreasing medication discrepancies when conducted at admission or discharge. However, it is difficult to assess the true impact of pharmacist-led medication reviews in specific high-risk populations, such as pediatric and geriatric populations.

In such a context, it is important to demonstrate the effectiveness of medication reconciliation as part of a standardized medication review process—in pediatric and elderly populations—on all-cause readmissions in a large randomized controlled clinical trial.

The aim of this study is to assess the impact of the pharmacist-led medication review on the rate of readmissions and/or death after hospital discharge and patient treatment satisfaction.

**Methods/design:**

The study is a randomized controlled clinical trial. A total of 1400 hospitalized patients will be randomized in two groups: (1) the experimental group (group receiving a pharmacist-led medication review) and (2) the control group (group receiving usual care). The pharmacist-led medication review process includes medication reconciliation, treatment review and medication liaison service. The primary endpoint will be the rate of readmissions and/or death at 30 days following initial hospitalization discharge. The secondary endpoints will be the rate of hospital readmission, the rate of emergency department visits, the rate of mortality, the number of consultations and patient treatment satisfaction at 30 days following initial hospitalization discharge.

**Discussion:**

A randomized controlled trial provides the most extensive evidence on the impact of pharmacist-led medication reviews on early hospital readmission for extreme age populations.

**Trial registration:**

Current Controlled Trials, NCT02734017. Registered on 4 May 2016.

**Electronic supplementary material:**

The online version of this article (doi:10.1186/s13063-017-1798-6) contains supplementary material, which is available to authorized users.

## Background

Early hospital readmission of patients after discharge is a public health problem. One major cause of hospital readmission is dysfunctions in integrated pathways between community and hospital care that can cause adverse drug events (ADE). An ADE is generally defined as a side effect (anticipated or unanticipated) of an administered medication; it may be due to an adverse drug reaction or a medication error [1]. Furthermore, the French ENEIS 2 study showed that 1.3% of hospital stays originated from serious ADE in 2009 [2]. Data from the literature show that older patients and children are at high risk of medication errors specifically at hospital admission and discharge [3–5].

In accordance with the World Health Organization (WHO) ("Assuring medication accuracy at transitions in care”) and French health authorities [6], developing programs on the securing patients’ medications is crucial through the course of care [7]. Indeed, transitions between community and hospital settings are at the highest risk of medication errors [3].

In this context, the pharmacist-led medication review may appear to be a way to improve patient safety. The medication review is a structured, critical examination of a patient’s medicines; the objective is to reach agreement with the patient regarding treatment to optimize the impact of medicines, minimize the number of medication-related problems and reduce waste [8]. When performed by pharmacists, this is called a pharmacist-led medication review.

The pharmacist-led medication review has been demonstrated to be an effective strategy to reduce medication discrepancies at admission and discharge [9, 10]. However, it is difficult to assess the real impact of the pharmacist-led medication review in specific high-risk populations, such as pediatric and geriatric populations.

Meta-analysis shows that pharmacist-led medication reviews have brought substantial reductions in the rate of readmission [10]. This endpoint is important because the pharmacist aims to improve reductions in medication errors as well as the transmission of information to the interfaces of the care system [11]. However, the studies included have certain limitations: (1) a low level of evidence, (2) the heterogeneity of experimental intervention (medication reconciliation associated or not with treatment review or medication service liaison) [12–20], (3) a different delay and different modalities in the endpoint assessment and (4) only one randomized controlled trial (RCT) performed in a pediatric population [12–16].

In such a context, it is important to demonstrate the effectiveness of a pharmacist-led medication review in high-risk populations (geriatrics and pediatrics) using a high level of evidence based on a large randomized controlled clinical trial. In our study, we hypothesized that this pharmacist-led medication review—which consists of (1) preventing medication errors at transitions (admission or discharge) through medication reconciliation, (2) analyzing and revising treatment, (3) informing and educating patients and (4) coordinating health professionals—may decrease hospital readmissions.

The primary objective of this study is to assess the impact of the pharmacist-led medication review on the rate of all-cause hospital readmissions and/or all-cause death at 30 days following initial hospitalization discharge. The secondary objectives are to assess the impact of the pharmacists-led medication review on the rate of all-cause readmission in care units, the rate of emergency department visits, the rate of mortality, the number of consultations post-discharge and patient treatment satisfaction.

## Methods/Design

### Design

**Fig. 1:**
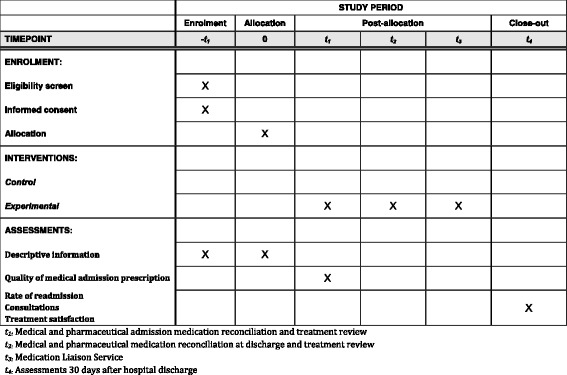
Template ConcReHosp Study

This is a prospective, randomized, controlled and open-label study comparing two patient care strategies: (1) the experimental group, receiving a pharmacist-led medication review; (2) the control group, receiving typical care. The open procedure is the only possible option because of the nature of the intervention, which requires that healthcare workers and participants not be blinded. The study protocol was designed using the recommendations of the Consolidated Standard of Reporting Trials (CONSORT) statement. The Standard Protocol Items: Recommendations for Interventional Trials (SPIRIT) checklist (Additional file 1) and figure (see Fig. 1) were used to prepare the study protocol.

### Partners

The sponsor of this study is the Assistance Publique-Hôpitaux de Marseille (AP-HM, France). This work is supported by institutional grants from the French 2014 National Program of Clinical Research (Programme de Recherche sur la Performance du Système de Soins). All details are provided in Table 1.

**Table 1:** French partners

Pharmacists	Center/department
Dr. Stéphane Honoré	Coordinating investigator, Pôle Pharmacie, Public Academic Teaching Hospital, Marseille
Dr. Pierre Bertault-Peres	Pôle Pharmacie, Public Academic Teaching Hospital, Marseille
Dr. Marie-Anne Estève	Pôle Pharmacie, Public Academic Teaching Hospital, Marseille
M. Pierre Renaudin	Pôle Pharmacie, Public Academic Teaching Hospital, Marseille
Dr. Clémence Tabélé	Pôle Pharmacie, Public Academic Teaching Hospital, Marseille
Dr. Florian Correard	Pôle Pharmacie, Public Academic Teaching Hospital, Marseille
Pediatric specialists	
Pr Michel Tsimaratos	Multidisciplinary Pediatric Care Unit, Public Academic Teaching Hospital, Marseille
Internal medicine geriatric specialists	
Pr Patrick Villani	Internal Medicine Geriatric Care Unit, Public Academic Teaching Hospital, Marseille
Dr Aurélie Daumas	Internal Medicine Geriatric Care Unit, Public Academic Teaching Hospital, Marseille
Dr Stéphane Gayet	Internal Medicine Geriatric Care Unit, Public Academic Teaching Hospital, Marseille
Methodologists	
Pr Pascal Auquier	Public health, Public Academic Teaching Hospital, Marseille
Dr Karine Baumstarck	Clinical Research Unit, Public Academic Teaching Hospital

### Recruitment of participants and study setting

The recruitment will be performed in a pediatric department and a geriatric and internal medicine department. The methodological support will be provided by the Clinical Research Unit (Unité d’Aide Méthodologique à la Recherche Clinique, AP-HM, France). The central pharmacy of AP-HM is in charge of overall coordination of the study and implementation of interventions.

### Inclusion and exclusion criteria

The details of the inclusion and exclusion criteria are provided in Table 2. The main inclusion criteria are (1) a pediatric patient hospitalized in a multidisciplinary pediatric care unit or (2) adult patient over 65 years old hospitalized in a geriatric and internal medicine post-emergency care unit where nearly all patients are admitted after an emergency room stay. The main exclusion criteria are patients whose care requires regularly/programmed re-hospitalization less than 30 days after discharge from the initial hospitalization.

**Table 2:** Selection criteria

Inclusion criteria
- Subject aged under 18 or over 65 years
- Subject hospitalized in the multidisciplinary pediatric care unit or internal medicine, therapeutics, post-emergency care unit, regardless of the reason for admission
- Subject with or without any comorbidity
- Living in France
- With national public funded health insurance
Exclusion criteria
- Patients whose care requires regular/programmed re-hospitalization less than 30 days after discharge from initial hospitalization.
- Vulnerable persons according to French law (pregnant women, adults under guardianship, persons deprived of liberty)

Participants will be recruited within 24 h after admission in the care service. When a new patient is eligible according to the selection criteria, a participant information sheet presenting the objectives of the study as well as procedures is provided and explained:

- To the adult patient or caregiver in the case of demented patients (Mini Mental State < 21);
- To the parents in the case of minor patients.

To reach the target sample, when a patient is admitted into the service, the physician performing the clinical examination of entry reviews the patient's inclusion criteria and considers whether the patient is eligible for study. He or she then informs the pharmacist of the study.

### Randomization

Computer-generated randomized lists will be drawn up before the beginning of the study using a permuted block design under the responsibility of the clinical research unit (AP-HM). The randomization will be stratified by center. The patients will be randomized to the control group or the experimental group using a computer-generated randomization schedule with a 1:1 allocation ratio. The open-label study is the only option because of the involvement of the patient in the medication reconciliation procedure.

## Experimental and control interventions

**Fig. 2:**
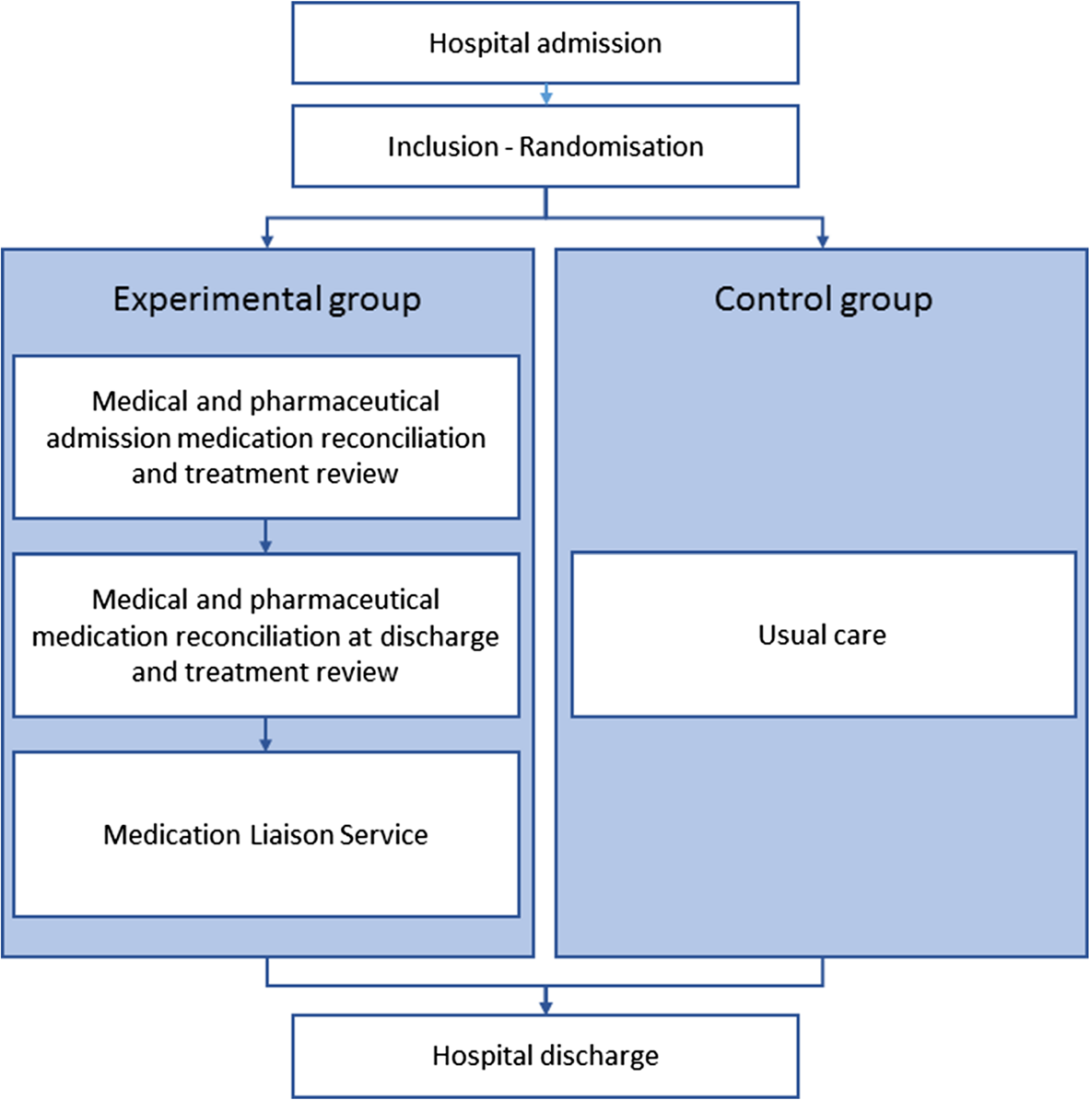
ConcReHosp study - flow of the intervention

Each patient included in the study will be randomized in one of the following two groups (see Fig. 2):

### Control group

In the control group, the pharmaceutical team is only involved in dispensing drugs, including standard pharmaceutical analysis prescription, during the hospital stay. At admission, the medical staff collects the list of current medications. At discharge, the medical team provides a prescription without intervention by the hospital pharmacy team. A report is sent to the community physician at the end of hospitalization.

### Experimental group

Participants in the experimental group receive a pharmacist-led medication review including the following: (1) a medical and pharmaceutical admission medication reconciliation and treatment review, (2) a medical and pharmaceutical medication reconciliation at discharge and treatment review and (3) medication liaison service.

#### Medical and pharmaceutical admission medication reconciliation and treatment review

Medication reconciliation (MedRec) at admission will be performed by a pharmacist or a pharmacy resident within 24 h after patient admission. On Saturdays and Sundays, admission MedRec will take place the following Monday morning, and for patients admitted on holidays, the MedRec will be performed the day after.

The admission MedRec first involves completion of the medication history, with research into information about patient treatment. Data from several sources should be gathered; previous medical and pharmaceutical records before admission or information from the patient him- or herself and/or the family or caregivers, the community pharmacist and physician should be gathered by phone. First, the pharmacist or a pharmacy resident interviews the patient, and then the interview is conducted with the community pharmacist. If there are insufficient data, the pharmacist should call the general practitioner or the specialist. Based on these data, it is possible to formalize the complete list of medications taken by the patient before hospitalization. Then, the list is compared to the admission prescription. Differences from the admission prescription are discussed between the hospital pharmacist and hospital physician. If these are intentional, the hospital physician is asked to note the reason in the medical record. In the case of medication errors, the hospital physician issues a revised prescription.

A pharmacist-led treatment review is systematically associated with MedRec. It consists of pharmaceutical analysis of the prescription.

#### Medical and pharmaceutical medication reconciliation at discharge and treatment review

The medical and pharmaceutical MedRec at discharge consists of comparing the medication history to the hospital discharge prescription to identify possible discrepancies. Differences between medication history and the hospital discharge prescription are discussed between the hospital pharmacist and hospital physician. The differences may be intentional, and if this is the case, the hospital physician notes the changes in the patient record. In the case of unintentional discrepancies (medication errors), the hospital physician issues a new prescription.

A pharmacist-led treatment review is systematically associated with MedRec. It consists of pharmaceutical analysis of the prescription.

#### Medication Liaison Service

At discharge, the medication liaison service includes the following:

- A comprehensive medication history contains the list of medications that the patient was taking prior to hospitalization and the list of new medications with comments, making it possible to understand the evolution of the treatment.
- Counseling: A session with the patient explaining the comprehensive medication history, the main adverse drug reactions, warning signs, biological monitoring, indications and an indication of the importance of drug adherence.
- A discharge letter faxed to the community pharmacist and general practitioner (community physician). This discharge letter will also present medications prescribed before and after hospitalization. Additional comments allow the community pharmacy and physician to understand the patient's revised treatment.

### Endpoints/Evaluation criteria

#### Primary endpoints

The primary endpoint is the rate of all-cause hospital readmission and/or all-cause death and/or emergency department visits occurring within 30 days after the patient discharge from initial hospitalization.

All-cause hospital readmission is defined as an unscheduled hospital stay in a medical or surgery department or intensive care unit, regardless of the reason for admission or duration of stay.

Death is defined as an occurrence of death between discharge and day 30.

Not considered are inpatient services, post-acute care and rehabilitation (PACR) or nursing homes.

#### Secondary endpoints

- The rate of all-cause hospital readmission, as defined in the primary endpoint, within 30 days after the patient discharge from the initial hospitalization.
- The rate of all-cause emergency department visits occurring within 30 days after the patient discharge from the initial hospitalization. This is the proportion of emergency department visits.
- The rate of all-cause mortality occurring within 30 days after the patient discharge from the initial hospitalization. This is the proportion of deaths.
- The number of consultations scheduled or not within 30 days post-hospitalization will be studied. Consultations are defined as interviews with the attending physician, a specialist physician or a nurse.
- Patient satisfaction with regards to its drug treatment will be measured using the standardized questionnaire “Satisfaction with Medicines Questionnaire (SatMed-Q®)” [21] validated in French [22]. It includes 17 items related to 6 different aspects: effectiveness, adverse effects, ease of use, patient's general opinion, treatment effect on daily life, quality of monitoring and information provided by health professionals. Answers are expressed on a 5-point Likert scale from "Not at all" to "Yes, a lot." There are six scores and an overall score (0 to 100). The evaluation will be conducted at 30 days post-hospitalization during the telephone follow-up.

### Follow-up and data collection

The evaluation will be performed at three different time points: baseline (T1), discharge at the hospital (T2) and 30 days after discharge from the hospital (T3). The study procedure and date collection are detailed in Table 3 and have been established as per the SPIRIT guidelines. A clinical research associate will collect the data with the clinical pharmacist. They will be obtained from the analysis of institutional documents of the medication reconciliation between admission and discharge and telephone follow-up documents 30 days after discharge.

**Table 3:** Study procedure

	T1	T2	T3
Consent	X		
Randomization	X		
Satisfaction		X	
All-cause hospital readmission			X
All-cause emergency department visits			X
All-cause mortality			X
Consultations			X

*T1* baseline, *T2* discharge at hospital, *T3* 30 days after discharge hospital

### Data protection

As regards the computerized processing of data relating to this project, which is intended for health research, it falls within the scope of legislative requirements, in particular the law of 9 August 2004, and will directly or indirectly identify the persons concerned.

### Statistical considerations

#### Sample size, power and statistical methods

The calculation of the required number of subjects was made from the primary endpoint, i.e., the rate of re-hospitalization and/or death.

Based on the literature, mainly a recent meta-analysis including the RCTs working on a pharmacist-led medication review [11], and data provided by the medical information service, the expected proportion of re-hospitalization and/or death in the standard group is 20% (worst case scenario). To detect a difference of at least 6% between the two groups (i.e., 14% re-hospitalization of the experimental group) given a two-sided significance level of 5% and 80% power, 1300 patients are needed (650 in each group). To prevent 10% dropout, a total of 1400 subjects should be included. The recruitment will be open for 18 months, and we expect to recruit about 75 patients per month.

#### Data analysis

The major principles of the analysis are reported below. However, a specific protocol of the analysis will be provided a second time before the initialization of the study (validated by the investigator coordinator, the person responsible for the analysis, and the biostatistician). The data will be analyzed using SPSS version 17.0 software. Statistical significance is defined as *p* < 0.05. The methodology is based on the Consolidated Standards of Reporting Trials Statement (CONSORT, http:// www.consort-statement.org/consort-statement/) [23].

The full analysis population (including all subjects who will be randomized and at least evaluated at the baseline) will be used in the primary analysis, and the per protocol population (including all subjects who will be randomized and will not have major protocol deviations) will be used in the secondary analysis to assess the robustness of the results. No interim analysis is planned. Demographic and baseline characteristics will be summarized for the two groups (‘standard’ and ‘experimental’ groups). Missing data will not be replaced.

Primary endpoint. The proportions of readmission and/or death at day 30 will be compared between the two groups (chi^2^ test or Fisher’s exact test). Proportions will be presented with 95% confidence intervals. Logistic regression will be performed to adjust potential confounding factors; variables relevant to the models will be selected based on their clinical interest and/or a threshold *p*-value ≤ 0.1 during univariate analysis. The center will be automatically entered into the model. The final models expressed the odd ratios and their 95% confidence intervals. The unadjusted analysis will be the primary analysis, and the adjusted analysis will be the complementary analysis.

Secondary endpoints. Qualitative variables will be compared between the two groups using the same procedure. The number of consultations and satisfaction scores will be compared between the two groups using Student’s *t* test or Mann-Whitney test. Multivariate approaches (logistic or linear regressions) will be performed using the same procedure.

### Data management and data quality

Quality assurance and control, under the responsibility of the promoter, will be conducted in accordance with Good Clinical Practice (decisions of 24 November 2006) to guarantee the integrity of the data collected and the protection of patients as well as to respect the protocol and legislation throughout the entire period of patient inclusion and follow-up by a clinical researcher mandated by the sponsor.

The nature and frequency of monitoring are established according to the definition of the level of monitoring according to the patient's risk and will depend on the number of patients included, the rhythm of the inclusions and the difficulties observed during the study (procedures validated by the Quality Working Group of the FHF Promotion, which determines the level of monitoring to be carried out according to the risk for the subject) (OECD Recommendation on the Governance of Clinical Trials, December 2012).

In this test, the level of monitoring is classified as "minimal" with a patient risk of type A. This will be to verify the consents. If one or more consents are not compliant, files will be monitored randomly.

### Ethical aspects, laws and regulations

This study is sponsored by the French Ministry of Health (PREPS 2014 no. 14–0330). The Assistance Publique-Hôpitaux de Marseille is the promoter and is in charge of all the administrative measures. As required by French law, all patients or their relatives will provide written informed consent to participate [24]. The French committees for data handling (CIL) approved the study. The study has been registered at ClinicalTrials.gov since March 2016 under the number NCT02734017.

## Discussion

To our knowledge, this is the first study to assess the effectiveness of pharmacist-led medication reviews in two extreme populations at high risk of medication errors, i.e., pediatric and geriatric populations. Moreover, the impact of pharmacist-led medication reviews on all-cause readmissions is not clear, and to our knowledge, there are no randomized controlled trials involving a large number of subjects [11].

In our study, we will evaluate patient satisfaction with their drug treatments. We hypothesize that a patient with a more precise explanation of his or her treatment will be more satisfied. Satisfaction being correlated with adherence, we believe this may decrease ADE [25].

We will use a randomized, open-label, controlled design, which is the most appropriate design to demonstrate the efficacy of pharmacist-led medication reviews in accordance with an Evidence-Based Medicine Working Group.

However, some issues related to the content of the protocol study should be discussed.

The open procedure is justified because the nature of intervention meant that personnel and participants could not be blinded. Indeed, the patients and the pharmacist are players in the intervention effect.

A cluster design should be proposed. Indeed, the experimental program is applied to the whole center and may affect all the individuals within it. To conform to the intervention application and to avoid contamination within the unit, cluster randomization may be advocated. However, this design requires the inclusion of several centers, and we have chosen to restrict the number of centers; we consider it easier to implement the experimentation in adherent centers, where the pharmacist teams and medical teams are working together, the best guarantee of the feasibility of the study. This risks minimizing the experimentation effect. Moreover, it is possible that our trial has less power because the pharmacist intervenes in both groups. However, it is not possible that the pharmacist does not intervene in the controlled group as required by law. Our tests therefore evaluate additional activities that have not yet shown their superiority.

The outcomes will be collected during a telephone follow-up 30 days after hospital discharge. We will call patients in the case of a patient who is older than 65 years, parents in the case of a minor patient or caregivers in the case of an elderly patient who is not able to consent. Moreover, if the patient reports a hospitalization or a visit to the emergency department during the 30 days post-discharge, a verification will be made by telephone call in the care unit.

## Trials status

At the time of manuscript submission (April 2016), the status of the trial is “open for participant recruitment.”

## Additional file

Additional file 1: SPIRIT 2013 Checklist: Recommended items to address in a clinical trial protocol and related documents*. (DOC 120 kb)

## Abbreviations

DGOS: Direction Générale de l’Offre de Soins
DGS: Direction Générale de la Santé
ENEIS: Enquête nationale sur les événements indésirables graves associés aux soins
HAS: Haute Autorité de Santé
PREPS: Programme de recherche sur la performance du système des soins
